# Chronic stress promotes colorectal cancer progression by enhancing glycolysis through β2-AR/CREB1 signal pathway

**DOI:** 10.7150/ijbs.79583

**Published:** 2023-04-02

**Authors:** Yunfeng Guan, Wang Yao, Hao Yu, Ying Feng, Yiyang Zhao, Xiangyang Zhan, Yan Wang

**Affiliations:** 1 Department of Medical Oncology, Shuguang Hospital, Shanghai University of Traditional Chinese Medicine, Shanghai, China; 2 Academy of Integrative Medicine, Shanghai University of Traditional Chinese Medicine, Shanghai, China; 3 Center of Urology, Shuguang Hospital, Shanghai University of Traditional Chinese Medicine, Shanghai, China

**Keywords:** chronic stress, colorectal cancer, glycolysis, β2-AR, CREB1

## Abstract

Colorectal cancer (CRC) is a common malignancy worldwide, and chronic stress has been considered as a significant risk factor for CRC. However, the role of chronic stress in CRC progression is unclear. The present study showed that pre-exposure to chronic stress facilitated CRC tumor growth in mice, and epinephrine promoted CRC cell proliferation *in vitro*. Metabolomics analysis revealed that chronic stress reshaped metabolic pathways to enhance glycolysis. Additional studies have shown that stress enhanced the expression levels of glycolytic-associated enzymes, including GLUT1, HK2 and PFKP. Mechanistically, chronic stress activated the β2-AR/PKA/CREB1 pathway, as a result, phosphorylated CREB1 transcriptional induced glycolytic enzymes expression. Furthermore, stress-induced cell proliferation and tumor growth could be reversed by administration of glycolysis inhibitor 2-deoxyglucose (2-DG) and β2-AR antagonist ICI118,551, respectively. Altogether, these findings define novel insights into the stress-induced epinephrine-mediated CRC progression from the point of view of tumor energy metabolism reprogramming and provide a perspective on targeting glycolysis as a potential approach in stress-associated CRC treatment.

## Introduction

Colorectal cancer (CRC) ranks third in terms of incidence, but second in terms of mortality worldwide 1. Along with changes of lifestyle and socioeconomic development, the incidence and mortality of CRC are steadily rising in many countries, including China 1, 2. Primary prevention and treatment remain the key strategies to reduce the increasing global burden of CRC. Therefore, further research is urgently needed to elucidate the specific underlying causal factors in the development of CRC.

Cancer patients usually experience a variety of chronic psychological distresses, including depression, anxiety, and fear 3, 4. These stressors serve as risk factor of a variety of cancer by facilitating tumor progression 5, 6. Previous studies have shown that chronic stress increased plasma catecholamine levels, including epinephrine and norepinephrine, and promoted tumor burden in ovarian carcinoma, breast cancer and gastric cancer 7-9. The development of CRC typically results from both genetic and environmental factors, as an extrinsic factor, chronic stress was also involved in the progression of CRC 10, 11. It was suggested that β-blocker treatment of patients suffering from CRC may lead to improved survival 12. Systemic stress has been shown to accelerate growth of CRC in immunocompromised mice 13. However, the detailed mechanism by which stress promotes the development of CRC is still not fully understood.

Cellular metabolism is deregulated in cancer to facilitate tumor survival and growth 14. A wealth of studies has detailed cell metabolic reprogramming and associated mechanism in the past decade. More recent studies have revealed that the properties of the tumor microenvironment (TME) can also influence metabolic programs 15. Chronic exposure to stress derived hormones changes in TME reshape metabolic processes of tumor cells to overcome nutrient limitation and sustain its malignant properties 9, 16, 17. For instance, chronic stress induced epinephrine promoted stem-like properties by lactate dehydrogenase A (LDHA) mediated metabolic rewiring to facilitate breast cancer progression 9. The initiation and development of CRC are a multi-step process and accompanied by metabolic alterations, such as enhanced glycolysis and lipogenesis 18. However, the role of stress on cellular metabolism in the progression of CRC has not been addressed.

Here, we revealed that chronic stress-induced epinephrine activated β2-AR exerted favorable effects on CRC development. Mechanistically, we verified a novel molecular basis that β2-AR/PKA/CREB1 signaling positively regulated glycolysis by increasing the expression of glycolytic enzymes transcriptionally. Importantly, the inhibition of glycolysis and β2-AR signaling suppressed chronic stress induced cell proliferation and tumor growth.

## Materials and Methods

### Cell Culture

Human colorectal cancer cell lines (HT-29, SW480, LoVo), human colon mucosal epithelial cell lines (NCM460) and mouse colorectal cancer cell lines (MC38, CT26) were purchased from Chinese Academy of Sciences cellbank (Shanghai, China) and preserved by our lab. Cells were cultured in Dulbecco's modified Eagle's medium (DMEM; Gibco, for MC38, HT-29 and NCM460), Leibovitz' L-15 medium (L-15; Gibco, for SW480) or Ham's F-12K (Kaighn's) medium (F12K; Gibco, for LoVo) supplemented with 10% (v/v) fetal bovine serum (FBS; Gibco, USA) and 1% penicillin/streptomycin (Gibco). The tumor isolated cells were cultured in DMEM as MC38. All cells were maintained at 37°C in an atmosphere containing 5% CO_2_. All cell lines were kept frozen in liquid nitrogen and after they were thawed, less than 30 passages were used for 3 months in the current experiments.

For *in vitro* chronic stress studies: The indicated cells were maintained in DMEM or L-15 supplemented with 2% FBS for 5 days with epinephrine (10 nM) treatment 9, then the cells were subjected to cell proliferation, colony formation, and metabolic studies. For pharmacological studies in figure 4 and dual luciferase assay, cells were maintained in 10% DMEM or F12K with indicated concentration of drugs as followed: epinephrine (10 μM), ICI118,551 (10 μM), H-89(10 μM) treatments.

### Reagents

Sucrose (#V900116), glucose (#G7021), and 4′,6-diamidino-2-phenylindole (DAPI, #D9542) were purchased from Merck. Epinephrine (#S2521) and oligomycin A (#S1478) were purchased from Selleck. ICI 118,551 (#HY-13951) and H-89 (#HY-15979A) were purchased from MCE, 2-deoxyglucose (2-DG; #A602241) was purchased from Bio Basic Inc. 2-(N-(7-Nitrobenz-2-oxa-1,3-diazol-4-yl)Amino)-2-Deoxyglucose (2-NBDG; #N13195) was purchased from Invitrogen. Deuterium oxide (D_2_O; #TL-zs-100g+tmsp) was purchased from Qingdao Tenglong. Antibodies against HK2 (#2867S), PFKP (#8164S), PKM2 (#4053S), LDHA (#3582S) and p-CREB1 (#9198S) were purchased from Cell Signaling Technology. Antibodies against tubulin (#11224-1-AP), ALDOA (#11217-1-AP) and CREB1 (#12208-1-AP) were purchased from Proteintech. Antibodies against GLUT1 (#ab115730), Ki-67 (#ab15580) and β2-AR (#ab182136) were purchased from Abcam.

### Mice

Six weeks old male C57BL/6 mice were purchased from Shanghai JSJ Laboratory Animal Co., Ltd. (Animal Quality Certificate: SCXK (Shanghai) 2021-0006) and maintained in a SPF-grade lab individually in cages for 3 days to adapt to the environment. The housing ambient temperature was between 22 and 25 ℃ with a humidity of 60% and a 12 h light/dark cycle. Mice were housed in groups of five per standard cage. Prior approval from the Ethics Committee of Shanghai University of Traditional Chinese Medicine was obtained (PZSHUTCM210507013).

### Chronic stress mouse model

Chronic restraint stress (CRS) was performed using previously published protocols 19. Stressed mice were restrained horizontally in modified 50 mL Falcon tubes with holes for ventilation from 9:00 AM to 15:00 PM for 14 consecutive days. Mice in the control group were left undisturbed for 14 days. Behavioral tests were performed following this procedure. Then, MC38 cells or tumor isolated cells (2×10^6^ in 100 μL PBS) were subcutaneously injected into the right axillary of each mouse. Tumors were measured using a sliding caliper every 2 days, and tumor volume was calculated by the formula: Volume = length × width × width /2. The subcutaneous tumor volume that reached 2000 mm^3^ in any mouse was considered as the end point. For drugs treatment, 2-DG (1 g/kg/2 days, dissolved in saline, i.p.) was administered 7 days after tumor cell injection, ICI 118,115 (25 µM/100 µL, dissolved in water, i.p.) was administered 1 day before tumor cell injection.

### Open field test

The open field test was performed in open boxes (50 × 50 × 40 cm) illuminated with white light in a quiet room. Mice were habituated in the testing room for 2 h before the start of experiment, then were placed onto the central zone of the open field boxes individually, and were allowed to explore in the test area for 5 min. The position and locomotion of each mouse were recorded and analyzed with an automatic detection system (Duoyi, Shanghai, China). The 50 cm square were divided into 16 equal squares by the system to analyze the behavior. The equipment was cleaned with 75% ethanol and paper towels before the next trial.

### Sucrose preference test

The sucrose preference test was performed as previously described 20. Briefly, the mice were housed individually with two water bottles for 2 days, in the next 2 days, mice were free access to two bottles filled with 1% sucrose solution. On the testing day, the mice were incubated with two bottles for 24 h, one with tap water and the other with 1% sucrose solution. The placement of the two bottles was switched after 12 h to eliminate the effect of drinking behavior. The volumes of consumed water and sucrose solution were recorded. Sucrose preference was calculated as the sucrose preference% = (sucrose intake/total intake) × 100%.

### Proliferation assay

Cell viability was detected using cell counting kit (CCK8, #CK04, Dojindo) according to the manufacturer's instruction. Briefly, cells were seeded in 96-well plate followed by the indicated drugs treatment, then, the cells were incubated for 1 h with 10% CCK8 (v/v) medium, absorbance was read at 450 nm with Cytation 3 (BioTek).

### Colony formation assay

Cells were plated in 6-well plate for 48 h, then, cells were treated with indicated reagents for 10 days. The colonies were fixed in 4% paraformaldehyde for 10 min and stained with 0.5% crystal violent for 10 min, the colonies were photographed and analyzed with ImageJ software.

### Glucose uptake and lactate production assay

The glucose uptake and lactate production assay were described previously 21. Briefly, cells were incubated with or without 10 μM 2-NBDG for 1 h at 37 ℃, then, cells were trypsinized and washed with PBS, resuspended cells were subjected to flow cytometer analysis with a FACSCanto Ⅱ (BD) to determine the uptake of 2-NBDG, the cells incubated without 2-NBDG were used as the negative control. Cells culture media were collected, and an aliquot of 540 μL of each sample with 60 μL of D_2_O was added to magnetic resonance tubes, and data were collected using a Bruker Ultrashield 400 MHz NMR magnet system (Bruker).

### ATP measurement

Intracellular ATP levels were assayed using the ATP assay kit (#S0026, Beyotime Biotechnology) according to the manufacturer's protocol. Briefly, the indicated cells were harvested in lysis buffer and centrifuged at 12000 g for 5 min to obtain the supernatant fraction. The supernatant were used to measure ATP with Cytation 3 (BioTek).

### Metabolomics

Metabolomics were performed as previously described with modification 22. Tumor tissue samples were homogenized in saline and centrifuged, the supernatants were mixed with extraction liquid (V_methanol_: V_acetonitrile_ = 4:1) at 1:4 volume ratio and vortexed, then the samples were incubated at -20 ℃ for 1 h and centrifuged at 13000 rpm for 15 min, the supernatants were evaporated using a vacuum concentrator, the dry pellets were reconstituted in extraction liquid (V_acetonitrile_: V_water_ = 4:1), after centrifugation at 12000 rpm for 10 min, supernatants were transfer to a fresh 2 mL LC/MS glass vial for the UHPLC-QQQ-MS analysis.

### Glycolysis assays

The extracellular acidification rates (ECARs) of the cells were measured using an XF96 extracellular flux analyzer (Seahorse Bioscience). Cells were plated in XF96 plates and then acclimatized at 37 °C for 1 h in XF Base Medium supplemented with 2 mM glutamine. Measurements were performed under basal conditions and in response to 10 mM glucose, 5 μM oligomycin and 100 mM 2-DG. Experiments using the Seahorse system were performed with the following assay conditions: 3 min of mixture, 3 min of waiting, and 3 min of measurement. Glycolysis and glycolytic capacity were analyzed according to manufacturer's instruction.

### RNA isolation and real-time quantitative PCR

Total RNA was extracted from cells using TRI reagent (#T9424, sigma-Aldrich), and complementary DNA was generated using the HiScript® III RT SuperMix (#R323, Vazyme) according to the manufacturer's protocol. Then, quantitative PCR reactions were performed using the ChamQ SYBR qPCR Master Mix (#Q331, Vazyme) with the 7500 Real-Time PCR System (Thermo Fisher). The β-actin mRNA expression level was used as a control. The primer sequences for RT-qPCR were listed in Table S1.

### Western blotting

Cells were lysed with egg lysis buffer (ELB) (150 mM NaCl, 100 mM NaF, 50 mM TrisHCl (pH 7.6), 0.5% NP40 and 1 mM PMSF) supplemented with protease inhibitors (#P1005, Beyotime). Equal amounts of protein were separated by sodium dodecyl sulfate-polyacrylamide gel electrophoresis (SDS-PAGE) and then transferred onto polyvinylidene difluoride membranes (Millipore), and immunoblotted with indicated antibodies. horseradish peroxidase (HRP)-conjugated anti-mouse immunoglobulin G (IgG) or anti-rabbit IgG antibodies were used as secondary antibodies. The immunoreactive products were detected using Chemiluminescent HRP Substrate (Millipore).

### Dual luciferase reporter assay

The luciferase assays were performed using the dual luciferase reporter assay kit (#RG088, Beyotime Biotechnology) according to the manufacturer's protocol. Briefly, the indicated promoter oligonucleotides were amplified and subcloned into the luciferase vector PGL3-Basic, then, the indicated firefly reporter plasmids and renilla reporter plasmid were co-transfected into MC38 cells followed by epinephrine (10 μM) treatment for 24 h. The cells were harvested and the luciferase activity was assayed using the dual-luciferase reporter assay system with SpectraMax ID5 (Molecular Devices), renilla luciferase activity was used as a control.

### Chromatin immunoprecipitation (ChIP) assay

ChIP assays were performed with the ChIP Assay Kit (#17-295; Merck) according to the manufacturer's instructions. Briefly, cells were crosslinked with 1% formaldehyde and neutralized using glycine, then cells were collected and sonicated. The DNA-protein complexes were immunoprecipitated with the antibody against p-CREB1, rabbit IgG was used as a negative control. DNA was recovered and subjected to RT-qPCR. The primers used were listed in Table S2.

### TCGA data analysis

The correlation between CREB1 and glycolytic genes from patients with colon adenocarcinoma and rectum adenocarcinoma and corresponding normal tissues were accessed through a web-based tool (GEPIA 23, http://gepia.cancer-pku.cn/detail.php).

### Immunohistochemistry (IHC)

Paraformaldehyde fixed tumor tissue samples were embedded in paraffin, cut into 5μm sections. Sections were then incubated with primary antibodies followed by incubation with secondary antibodies. immunoreactivity was detected using 3,3′-diaminobenzidine tetrachloride (DAB). Images were recorded using M8 Microscope and Scanner (Precipoint). Then, positively stained areas were measured by cognitionMaster software.

### Immunofluorescence

Cells were seeded on microscope coverslips and fixed with paraformaldehyde. Then, the cells were washed with PBS and blocked with blocking buffer (3% BSA and 0.2% Triton X-100 in PBS) and incubated with the appropriate primary antibodies. The cells were then washed with washing buffer (0.2% BSA and 0.05% Triton X-100 in PBS) and incubated with Alexa Fluor-conjugated secondary antibodies (#A27034, Thermo). The cells were stained with DAPI before the cells were mounted and imaged using SP8 microscope (Leica). The mean fluorescence intensity (MFI) and subcellular MFI were measured by ImageJ (National Institutes of Health).

### Quantification and statistical analysis

For cell experiments study, sample size was determined to be adequate based on the magnitude and consistency of measurable differences between groups, usually the number is 3 or more. For the mouse model study, no statistical methods were used to predetermine sample size, which was determined based on previous experimental observations. Sample size for each experiment is indicated in the figure legend.

All statistical analyses were performed using GraphPad Prism 8 (La Jolla, CA, USA). Data are presented as the means ± SEM of at least three independent experiments unless otherwise specified. Significant differences between means were determined with two-tailed Student's *t*-tests or analysis of variance (ANOVA) followed by Tukey's post hoc test unless otherwise specified.

## Results

### Chronic stress promotes colorectal cancer progression

**Figure 1:**
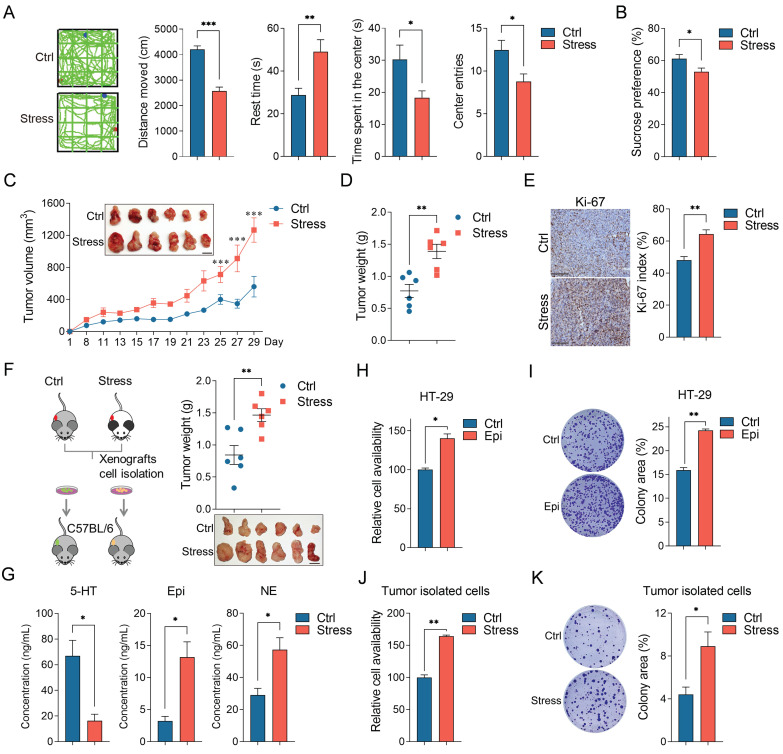
** Chronic stress promoted colorectal cancer development**. **(A)** Typical locomotion tracks (green lines) of control (Ctrl) mice and mice subjected to chronic stress (Stress) in open field tests (left). Distance moved, rest time, time spent in the center and center entries were compared between control (n=9) and stress (n=9) groups (right). **(B)** Sucrose preference test was conducted between control and stress groups after completion of the chronic stress paradigm (n=7). **(C and D)** Chronic stress promotes xenograft tumor growth. MC38 cells were injected subcutaneously into control and stressed C57BL/6 mice to form xenograft tumors (n=6). Tumor growth curve, representative xenograft tumor images (**C**) and tumor weights (**D**) are shown, Scale bar, 1 cm. **(E)** The expression levels of Ki-67 in tumor sections were evaluated by immunohistochemistry and Ki-67 positive cells were indicated as Ki-67 index. Scale bar, 100 μm. **(F)** Chronic stress education promoted xenograft tumor growth. The xenografts of control and chronic stress groups were dissociated and cultured, then injected subcutaneously into C57BL/6 mice (n=6), representative xenograft tumor images and tumor weights are shown, Scale bar, 1 cm. **(G)** The levels of epinephrine, norepinephrine and serotonin in control and chronic stress groups were determined by LC-MS. **(H-K)** Chronic stress promoted colorectal cells proliferation and colony formation. Control and epinephrine (Epi, 10 nM) treated HT-29 cells, cells isolated from control and chronic stress xenografts were subjected to CCK8 assays (**H, J**) and colony formation assay (**I, K**). The data are represented as the means ± SEM of at least three independent experiments. *, p < 0.05; **, p < 0.01; ***, p < 0.001.

Depression is a risk factor for the incidence and death of cancer 24, 25, to investigate the potential promoting role of chronic stress on colorectal cancer development, we took advantage of chronic restraint stress models as described previously 11 (Figure S1A). Repeated exposure of male C57BL/6 mice to persistent restraint for two weeks causes depression-like behaviors, which manifest as diminished total locomotion, time spent in the center and center entries, increased rest time in the open field test, reduced sucrose preference in the sucrose preference test, and body weight loss (Figure 1A, 1B and S1B). We determined the effect of chronic stress on colorectal cancer progression in mice inoculated with MC38 cells subcutaneously. Chronic stress in mice caused an increase in the tumor growth and tumor weight relative to non-stressed controls (Figure 1C and 1D). Histopathological examination suggested that chronic stress enhanced the Ki-67 index, a marker of cell proliferation (Figure 1E). To further study the effect of chronic stress on tumor growth, we isolated the tumor cells by enzymatic digestion from the control and stressed mice and reinoculated in normal mice, the weight of tumor from the stressed cells is remarkably higher than that from the control cells (Figure 1F), indicating that the chronic stress remodels the tumor cell growth trait.

Moreover, the adrenal glands of the chronic stress mice were clearly larger than that of the control mice, suggesting that the HPA axis remained active in the chronic stress state (Figure S1C). Next, we examined the concentration of the major adrenal stress hormones, including epinephrine, norepinephrine and serotonin (5-HT) in tumor tissues 26, 27, compared to the control group, the levels of epinephrine and norepinephrine were increased and the level of 5-HT was decreased in the chronic stress group (Figure 1G). As epinephrine is the most significantly upregulated hormones which is closely related to depression and tumorigenesis 28, so we accessed the roles of epinephrine in CRC cell lines, cell viability and colony formation ability were obviously enhanced with epinephrine exposure (Figure 1H, 1I, S1D and S1E). Consistently, stressed tumor cells presented enhanced cell viability and colony formation ability compared with the control tumor isolated cells (Figure 1J and 1K). Collectively, these data demonstrate that chronic stress promotes colorectal cancer growth *in vivo* and *in vitro*.

### Chronic stress induces metabolic reprogramming and enhances glycolysis

**Figure 2:**
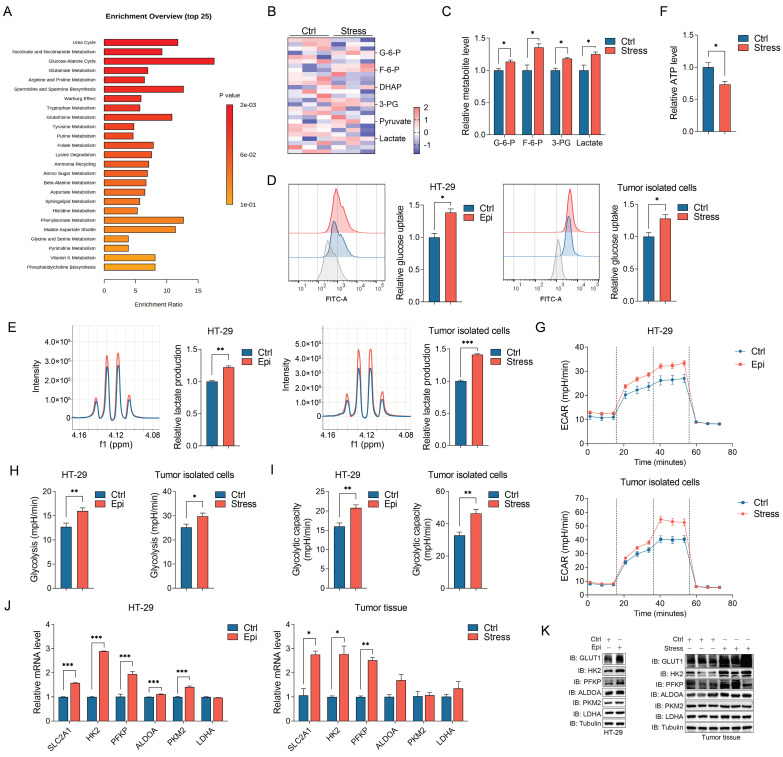
** Chronic stress increased glycolysis in CRC development**. **(A)** The xenografts of control and chronic stress groups were subjected to untargeted metabolomics analysis, and the metabolites were further conducted with metabolic pathway enrichment analysis. **(B and C)** Heatmap represented the metabolites of glycolysis that subjected to targeted metabolomics analysis (**B**), and significant different metabolites were shown (**C**). **(D)** The indicated cells incubated with 2-NBDG were analyzed with flow cytometer to determine the glucose uptake rate, the gray peak represented the cells incubated without 2-NBDG which were used as the negative control, and the representative FACS analysis were shown (left). **(E)** The supernatant of indicated cells were collected and subjected to lactate level determination, and the representative NMR analysis were shown (left). **(F)** The ATP level of control and stress groups were measured. **(G-I)** Seahorse analysis were conducted to determine the ECAR in indicated cells, the glycolysis and glycolytic capacity were shown. **(J)** The indicated gene expression levels in control and stress groups cells (left) and tumor tissues (right) were determined by RT-qPCR. **(K)** The indicated protein expression levels in control and stress groups cells (left) and tumor tissues (right) were determined by western blot. The data are represented as the means ± SEM of at least three independent experiments. *, p < 0.05; **, p < 0.01; ***, p < 0.001.

Chronic stress induced cellular metabolic reprogramming in cancers 29. To investigate how chronic stress contributes to CRC progression, we determined the metabolites changes in control and stressed tumors with metabolomics study, metabolic pathway enrichment analysis indicated that chronic stress promoted metabolic adaption (Figure 2A). Among these enriched metabolic pathways, glycolysis draws our attention as we noticed that the culture media of stressed cells turned yellow much faster than that of the control cells (Figure S2A), we hypothesized that this phenotype might due to the lactate acidosis. Aggressive tumors cells exert high glucose consumption and lactate production which can lead to the pH change 30, 31. Hence, we accessed the glycolytic metabolites alteration in control and stressed tumors with targeted metabolomics analysis, we found a significant increase in the levels of glycolytic metabolites in stressed tumors compared with control tumors (Figure 2B and 2C). Moreover, epinephrine treated and stressed cells (herein, both epinephrine treated cells and stressed cells isolated from tumors were referenced as stressed cells) increased glucose consumption and lactate production and decreased cellular ATP level compared with that of control cells (Figure 2D-2F). We next measured extracellular acidification rate (ECAR) which indicated the real time alteration of glucose metabolism in CRC cells, and found that stressed cells had an increased glycolytic capacity and higher glycolytic rate than that of control cells (Figure 2G-2I). These data suggest that stress reprogramed CRC cell metabolism to favor aerobic glycolysis.

To further gain insight into the regulatory role of chronic stress in glycolysis, the expression of the glycolysis-associated enzymes in CRC cells were examined, including GLUT1, HK2, PFKP, ALDOA, PKM2 and LDHA. We observed that the expression levels of GLUT1 (SLC2A1) and the glycolytic rate-limiting enzymes HK2 and PFKP were increased in response to stress both in mRNA and protein levels in CRC cells and tumor tissues (Figure 2J, 2K, S2B and S2C). Together, these findings illustrate that chronic stress increased glycolysis might through inducing the expression of glycolytic enzymes.

### Glycolysis blockade significantly suppresses CRC progression under chronic stress

**Figure 3:**
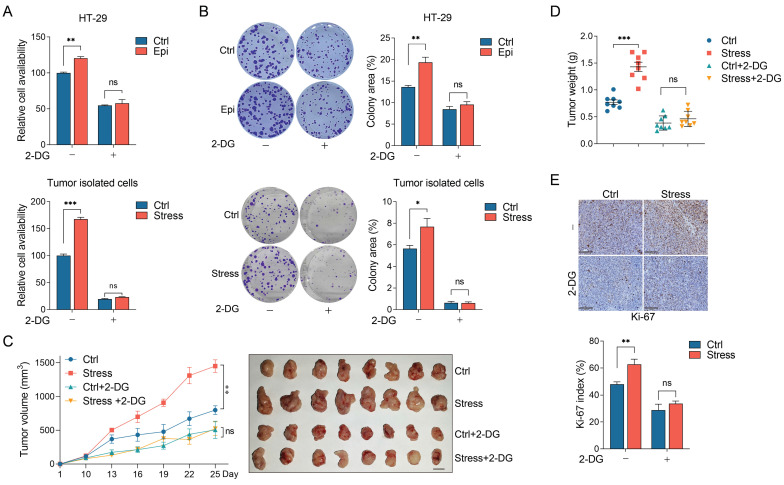
** Chronic stress enhanced glycolysis to promote CRC tumor growth. (A and B)** 2-DG treatment abolished chronic stress promoted colorectal cells proliferation and colony formation. Control and epinephrine (10 nM) treated HT-29 cells, cells isolated from control and chronic stress xenografts with or without 2 mM 2-DG treatment were subjected to CCK8 assays (**A**) and colony formation assay (**B**). **(C and D)** 2-DG administration blocked chronic stress promoted xenograft tumor growth. MC38 cells were injected subcutaneously into control and chronic stressed C57BL/6 mice to form xenograft tumors (n=8) with or without 1 g/kg 2-DG treatment. Tumor growth curve, representative xenograft tumor images (**C**) and tumor weights (**D**) are shown, Scale bar, 1 cm. **(E)** The expression levels of Ki-67 in tumor sections were evaluated by immunohistochemistry and Ki-67 positive cells were indicated as Ki-67 index. Scale bar, 100 μm. The data are represented as the means ± SEM of at least three independent experiments. *, p < 0.05; **, p < 0.01 and ***, p < 0.001, and ns, no significance.

We then tested whether glycolysis plays a role in chronic stress promoted CRC progression. It was demonstrated that the stressed CRC cells showed enhanced cell viability and colony formation ability compared with the control cells. However, when the control and stressed cells were treated with 2-DG (a glycolysis inhibitor) simultaneously, 2-DG treatment decreased the cell viability and colony formation ability in the control cells, importantly, it entirely reversed the cell proliferation promoting-effect in stressed cells compared with the control cells (Figure 3A and 3B).

To explore whether chronic stress promotes CRC tumor growth through glycolysis, we administrated the control and chronic stress tumor-bearing mice with 2-DG at day 8 after MC38 incubation subcutaneously. Consistently, chronic stress promoted tumor growth and enhanced tumor weight compared with the control group, however, 2-DG treatment not only suppressed tumor growth and tumor weight gain in control group, but also reversed the tumor-promoting effect of chronic stress (Figure 3C and 3D). We also determined the Ki-67 index with immunohistochemistry, compared with chronic stress group, 2-DG administration blocked the promoting effect of chronic stress on Ki-67 index (Figure 3E). Taken together, these results support the hypothesis that chronic stress promoted CRC progression through glycolysis.

### Chronic stress activates β2-AR/CREB1 signaling pathway

**Figure 4:**
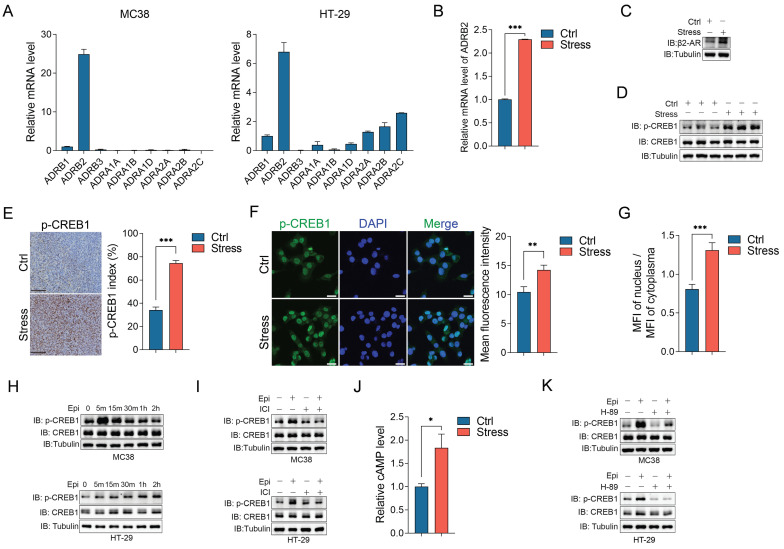
**Chronic stress activated β2-AR/CREB1 signaling pathway. (A)** The mRNA expression levels of 9 adrenoreceptors in MC38 and HT-29 cells were detected by RT-qPCR.** (B and C)** The mRNA expression level and protein expression level of β2-AR in control and stress groups were detected by RT-qPCR (**B**) and western blot (**C**), respectively.** (D)** The phosphorylation of CREB1 in xenografts was detected by western blot. **(E)** The phosphorylation of CREB1 was evaluated by immunohistochemistry and p-CREB1 positive cells were indicated as p-CREB1 index. Scale bar, 100 μm. **(F)** The phosphorylation levels of CREB1 in control and stressed cells were evaluated by immunofluorescence and quantified as MFI. Scale bar, 20 μm. **(G)** The MFI of nuclear and cytosolic phosphorylated CREB1 in control and stressed cells were evaluated by immunofluorescence and quantified as MFI ratio. **(H)** The phosphorylation of CREB1 in MC38 and HT-29 cells followed by 10 μM epinephrine treatment at indicated time were detected by western blot. **(I)** The effects of β2-AR antagonist ICI 118,551 (10 μM, pretreated 2 h) on epinephrine (10 μM) mediated CREB1 phosphorylation were detected by western blot in MC38 and HT-29 cells.** (J)** The cAMP levels of control and stressed xenografts were determined by LC-MS. **(K)** The effects of PKA inhibitor H-89 (10 μM, pretreated 2 h) on epinephrine (10 μM) mediated CREB1 phosphorylation were detected by western blot in MC38 and HT-29 cells. The data are represented as the means ± SEM of at least three independent experiments. *, p < 0.05; **, p < 0.01 and ***, p < 0.001.

Catecholamines, including epinephrine and norepinephrine, act on effector tissues through the adrenergic receptors (ARs) which are widely expressed in multiple tissues, including cancer tissues 26, 32. We detected the mRNA levels of 9 ARs in CRC cell lines, including HT-29, SW480, LoVo, MC38 and CT26, and human colon mucosal epithelial cell lines NCM460, consistent with the report that β2-adreneric receptor (β2-AR) is the major mediator for chronic stress mediated cancer development 33, we observed that β2-AR (ADRB2) is the dominant expressing ARs in these CRC cells (Figure 4A and S3A). In addition, chronic stress significantly upregulated the β2-AR mRNA and protein expression levels compared with the controls (Figure 4B and 4C). cAMP response element-binding protein 1 (CREB1) is a critical mediator for stress-induced cascade 34, thus, we determined its expression and activation under chronic stress. Although chronic stress barely affected the total protein levels of CREB1, it obviously upregulated the phosphorylation level of CREB1 compared with the control tumors (Figure 4D). Moreover, the immunohistochemistry and immunofluorescence assays of phosphorylated CREB1 further supported the role of chronic stress in activating CREB1 (Figure 4E, 4F and S3B). As CREB1 plays a central role in transcriptional regulation, subcellular localization of phosphorylated CREB1 also confirmed the activation of CREB1 under stress condition (Figure 4G).

Next, we detected whether chronic stress activates CREB1 through β2-AR. We firstly detected the role of epinephrine on CREB1 activation, epinephrine treatment clearly induced CREB1 phosphorylation in a time dependent manner (Figure 4H and S3C). Treatment with the specific β2-AR antagonist ICI 118,551 (ICI) abolished epinephrine induced CREB1 phosphorylation (Figure 4I and S3D). Previous metabolites analysis showed that the level of cAMP was increased in stressed tumors compared with the controls (Figure 4J), cAMP could activate protein kinase A (PKA) which can phosphorylate various kinases, such as CREB1, Raf, GSK3 35. We then treated CRC cells with PKA inhibitor H-89 which blocked the phosphorylation of CREB1 induced by epinephrine (Figure 4K and S3E). These results indicated that chronic stress activates β2-AR/cAMP/PKA/CREB1 signaling pathway in CRC cells.

### Chronic stress promotes CRC progression through β2-AR/CREB1 pathway

**Figure 5:**
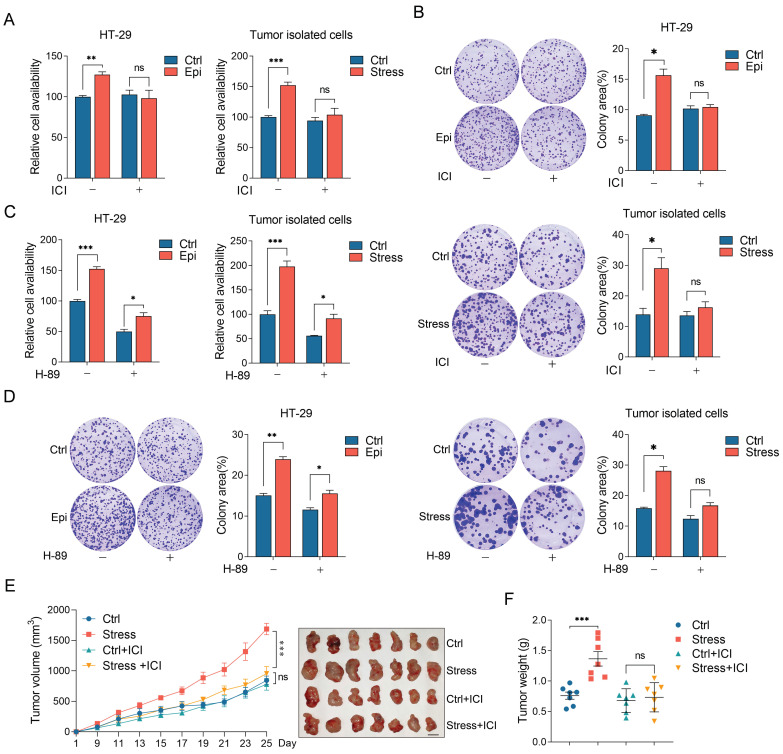
** Chronic stress promoted CRC development through β2-AR/CREB1 signaling pathway**. **(A and B)** The effects of ICI 118,551 on stressed CRC cell proliferation were detected by CCK8 assay (**A**) and colony formation assay (**B**) in HT-29 and tumor isolated cells. **(C and D)** The effects of H-89 on stressed CRC cell proliferation were detected by CCK8 assay (**C**) and colony formation assay (**D**) in HT-29 and tumor isolated cells. **(E and F)** ICI 118,551 administration blocked chronic stress promoted xenograft tumor growth. MC38 cells were injected subcutaneously into control and stressed C57BL/6 mice to form xenograft tumors (n=7) with or without ICI 118,551 treatment. Tumor growth curve, representative xenograft tumor images (**E**) and tumor weights (**F**) are shown, Scale bar, 1 cm. The data are represented as the means ± SEM of at least three independent experiments. *, p < 0.05; **, p < 0.01 and ***, p < 0.001, ns, no significance.

To explore whether the effect of chronic stress on CRC progression is mediated by β2-AR/CREB1 pathway, we analyzed the role of β2-AR on CRC cells isolated from tumors or treated with epinephrine. Consistently, the stressed cells showed a significant increase in cell viability and colony formation ability compared with the control cells. However, ICI treatment abrogated the effect of stress on cell viability and colony formation ability, but barely affected that of the control cells (Figure 5A and 5B). We then evaluated the role of PKA on chronic stress promoted cell proliferation and colony formation. Cell viability assays and colony formation assays revealed that H-89 evidently suppressed the effect of stress on cell viability (Figure 5C and 5D).

Next, we examined the role of β2-AR on CRC tumor growth. Control and chronic stress tumor-bearing mice treated with ICI before MC38 incubation subcutaneously. Consistently, chronic stress promoted tumor growth and enhanced tumor weight compared with the control group. However, ICI treatment suppressed tumor growth and tumor weight gain induced by chronic stress (Figure 5E and 5F). Collectively, these data showed that chronic stress activated β2-AR signaling to promote CRC progression.

### Chronic stress facilitates CREB1 transcriptional regulation of glycolysis

**Figure 6:**
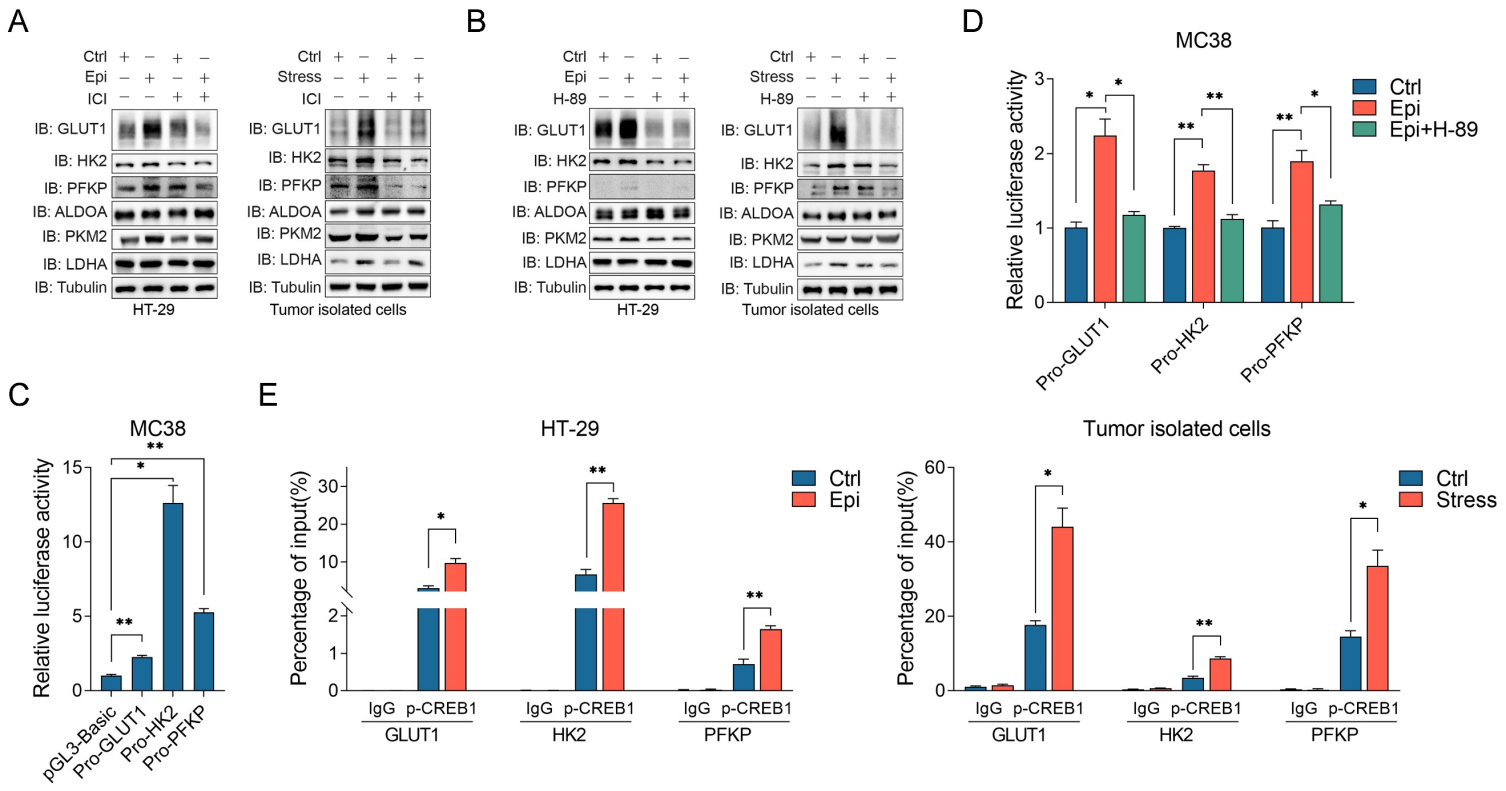
** Chronic stress elevated glycolytic enzymes expression via β2-AR/CREB1 signaling pathway**. **(A and B)** The effects of ICI 118,551 (10 μM) (**A**) and H-89 (10 μM) (**B**) treatment on chronic stress induced glycolytic enzymes expression were detected by western blot, respectively. **(C)** The luciferase activities of the promoters of GLUT1, HK2 and PFKP were determined in MC38 cells. **(D)** Epinephrine (10 μM) increased the luciferase activities of the promoters of indicated glycolytic enzymes. A luciferase assay was performed in MC38 cells with indicated drugs treatment.** (E)** p-CREB1 binds to the promoters of indicated glycolytic enzymes. ChIP assays were performed in HT-29 and tumor isolated cells. The data are represented as the means ± SEM of at least three independent experiments. *, p < 0.05; **, p < 0.01.

β2-AR signaling activation could lead to reprogramming of cell glucose metabolism 36. Thus, we determined whether chronic stress activated β2-AR signaling participated in the regulation of glycolysis. It was demonstrated that chronic stress promoted GLUT1, HK2 and PFKP expression, whereas ICI treatment impaired chronic stress induced glycolytic enzymes expression (Figure 6A). Moreover, H-89 treatment also abolished the effect of chronic stress on GLUT1, HK2 and PFKP expression (Figure 6B), indicating that β2-AR signaling is required for chronic stress induced glycolysis.

Previous report showed that CREB1 could potential regulated metabolism-related gene expression, including several critical glucose metabolism genes 37, suggesting that β2-AR/CREB1 signaling may directly regulate glycolytic enzymes expression transcriptionally. Hence, we constructed several promoter subclones for the luciferase assay (Figure 6C). As shown, epinephrine treatment effectively increased the activities of the GLUT1, HK2 and PFKP promoters. However, H-89 treatment blocked the effect of epinephrine on the increase of the luciferase activities (Figure 6D). To further verify this hypothesis, by performing the chromatin immunoprecipitation (ChIP) assay, we found that phosphorylated CREB1 could directly bind to the promoters of GLUT1, HK2 and PFKP in both control and stressed cells, whereas chronic stress enhanced the recruitment of phosphorylated CREB1 to the promoters of GLUT1, HK2 and PFKP when compared with the control cells (Figure 6E). These findings demonstrate that CREB1 directly binds to glycolytic enzymes promoters and transactivated their expression under chronic stress.

To evaluate the relevance of β2-AR/CREB1 signaling with glycolysis, we analyzed the relationship between CREB1 and glycolytic genes expression through the accessible TCGA data sets of COAD and READ, and we discovered that CREB1 is positive correlated with GLUT1 (SLC2A1), HK2, PFKP (Figure S4A-S4C). These results support that β2-AR/CREB1 signaling is involved in the regulation of glycolysis.

## Discussion

**Figure 7:**
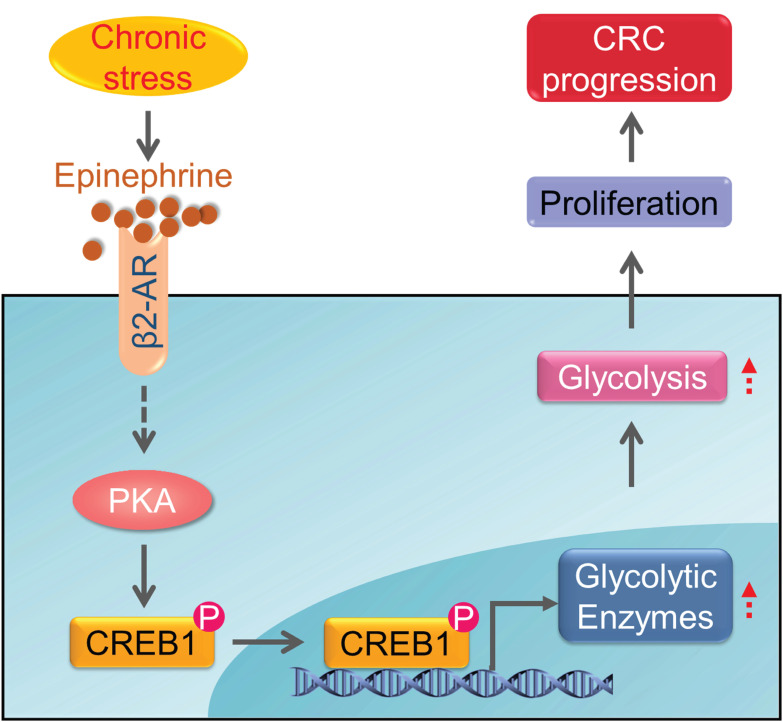
**Schematic diagram of the chronic stress/β2-AR/PKA/CREB1 axis through regulating glycolysis in CRC progression**. Chronic stress stimulates the elevation of epinephrine. Epinephrine interacts with the adrenoreceptor β2-AR to activate PKA which lead to the phosphorylation of CREB1. Then, phosphorylated CREB1 transcriptional activate glycolytic enzymes expression to promotes glycolysis. As a result, epinephrine activates glycolysis in CRC cells and promotes CRC development.

The common feature of chronic stress in cancer development is the aberrantly persistent activation of the hypothalamic-pituitary-adrenal axis and sympathetic nervous system, leading to the enhanced production of plasma catecholamines, including epinephrine and norepinephrine. In addition, cancer diagnosis, surgery and chemotherapy are usually extremely stressful events for the patients, causing long-term psychological distress, which will facilitate the deterioration of cancer 17, 28, 38. Here, we provide evidence that chronic stress could lead to depression-like behaviors and facilitate CRC tumor growth. Additionally, we found that chronic stress induced epinephrine activates β2-AR signaling to empower colorectal cell aggressive property by rewiring cellular metabolism. Furthermore, we demonstrated that β2-AR activates PKA/CREB1 signaling, leading to the phosphorylation of CREB1 which transcriptional regulates the expression of glycolytic enzymes (Figure 7). These findings expand our understanding of the role of metabolic remodeling in stress associated cancer progression and provide translational potential by blocking stress-induced epinephrine signaling pharmacologically to prevent tumor development.

Emerging evidence has pointed to chronic stress as a critical regulator in the metabolic reprograming that contributes to the growth of a variety of malignancies 9, 39, 40. Glycolysis is an important cellular process that plays a key role in tumor development. Using metabolites analysis, we found that chronic stress induced metabolic rewiring, and specifically, chronic stress enhanced glycolytic capacity by promoting glucose uptake and lactate production and extracellular acidification rates (Figure 2A-2I). GLUT1 and glycolytic enzymes were usually overexpressed or possessed enhanced enzyme activities to maintain aggressive properties of cancer cells by satisfying the high demand of energy source 41, 42. Consistently, we showed that chronic stress promoted the expression levels of glycolytic enzymes, including GLUT1, HK2 and PFKP (Figure 2J, 2K, S2B and S2C). Meanwhile, HK2 and PFKP are the rate-limiting enzymes in the process of glycolysis 42. Our current study demonstrates that glycolysis blockage indeed abrogated the tumor-promoting effect of chronic stress (Figure 3). These results indicated that chronic stress rewired cellular metabolism by preference targeting the genes that are critical for tumor progression.

The role of psychological state in tumor progression is supported by previous clinical study, which indicated that the significantly improved emotional functioning is correlated with cancer treatment 38. Catecholamines activated β2-AR that expressed on the surface of tumor cells is an essential step for tumor growth and progression in various stress models 36. Although β2-AR signaling likely promotes tumorigenesis through both the direct and indirect effects on tumor cells. Here, we showed that β2-AR is the prominent adrenergic receptor that expressed on the surface of CRC cells, and β2-AR blockage abrogated stress mediated CRC cell proliferation and tumor growth (Figure 5). Chronic stress induced catecholamines activate the cAMP/PKA signaling pathway through β2-AR, causing activation of a series of transcription factors, including CREB1 and NF-κB which lead to cell proliferation 8. We found that stress indeed increased cAMP level and PKA/CREB1 activation (Figure 4). Previous research indicated that several genes encode critical enzyme in glucose metabolism are putative CREB1 target genes, such as HK2, PFK1 and PKM2, and CREB1 mediated glycolysis phenotype in cancer-associated fibroblasts by transcriptional upregulating the expression of PDK4 and LDHB 37. Here, we found that stress-activated CREB1 transcriptional regulated the expression of GLUT1, HK2 and PFKP (Figure 6), resulting in an energy metabolism switch in CRC cells. Thus, the β2-AR/PKA/CREB1 pathway aroused by stress enhanced the expression of GLUT1, HK2 and PFKP and promoted glycolysis mediated CRC progression.

Previous research showed that psychological factors promoted stem-like properties in breast cancer cells and their tumorigenic potential 9. Moreover, paternal psychological stress exerts an intergenerational effect on modifying epigenetic signature and regulating glucose metabolism in offspring 43. These researches implied the long-term effect of chronic stress on physical traits of tumor cells. Here, by analyzing the cell proliferation properties of tumor cells from control and stressed tumors, we found that tumor cells from stressed mice grew faster than that from control mice (Figure 1F, 1J and 1K). In addition, tumor cells from stressed mice possessed enhanced glycolytic capacity when compared with the cells from control mice (Figure 2H, 2I, S2B and S2C). Furthermore, administrated with 2-DG, ICI118,551 and H-89 blocked the aggressive properties of cells from stressed mice (Fig 3A, 3B and 5A-5D). Epigenetic mechanism including DNA methylation and histone modification which collectively could provide a non-genetic molecular legacy of prior environmental exposures in offspring 44. It is reported that the microenvironmental changes enabled the epigenetic alterations and expression of key glycolytic enzymes in cancer-associated fibroblasts 30. Hence, the growth-promoting phenotype of cells from stressed mice may be attributed to the epigenetic changes by long-term chronic stress and high levels of catecholamines in the tumor microenvironment.

In summary, this study on CRC exemplifies the important role of epinephrine reshaped metabolism in the development of cancer. In the case of CRC, we have shown the crucial role at stage of CRC progression for elevated catecholamine levels. Our findings suggest that blocking β2-AR or glycolysis represents a promising preventive or therapeutic strategy for CRC. Furthermore, our study indicates the potential effect of chronic stress on long-term changes in tumor cells beyond metabolic rewiring.

## Supplementary Material

Supplementary figures and tables.

## Abbreviations

2-DG: 2-deoxyglucose
2-NBDG: 2-(N-(7-Nitrobenz-2-oxa-1,3-diazol-4-yl)Amino)-2-Deoxyglucose
5-HT: serotonin
ADRB2: Adrenoceptor beta 2
ALDOA: Fructose-bisphosphate aldolase A
ANOVA: Analysis of variance
ARs: Adrenergic receptors
ATP: Adenosine triphosphate
cAMP: Cyclic adenosine monophosphate
ChIP: Chromatin immunoprecipitation
COAD: Colon adenocarcinoma
CRC: Colorectal cancer
CREB1: cAMP responsive element binding protein 1
CRS: Chronic restraint stress
D_2_O: Deuterium oxide
DAB: 3,3'-diaminobenzidine tetrachloride
DAPI: 4',6-diamidino-2-phenylindole
DMEM: Dulbecco's modified Eagle's medium
DNA: deoxyribonucleic acid
ECAR: Extracellular acidification rates
ELB: Egg lysis buffer
F12K: Ham's F-12K (Kaighn's) medium
FBS: Fetal bovine serum
GLUT1: Glucose transporter type 1
GSK3: Glycogen synthase kinase 3
HK2: Hexokinase 2
HPA: Hypothalamic-pituitary-adrenal
HRP: Horseradish peroxidase
IgG: Immunoglobulin G
IHC: Immunohistochemistry
L-15: Leibovitz' L-15 medium
LC/MS: Liquid chromatography mass spectrometry
LDHA: lactate dehydrogenase A
LDHB: lactate dehydrogenase B
MFI: mean fluorescence intensity
mRNA: Messenger ribonucleic acid
NF-κB: Nuclear factor kappa B
PBS: Phosphate-buffered saline
PDK4: Pyruvate dehydrogenase kinase 4
PFKP: Phosphofructokinase, platelet type
PKA: Protein kinase A
PKM2: Pyruvate kinase, isoform M2
PMSF: Phenylmethanesulfonyl fluoride
Raf: Raf-1 proto-oncogene
READ: Rectum adenocarcinoma
RT-qPCR: real-time quantitative polymerase chain reaction
SDS-PAGE: Sodium dodecyl sulfate-polyacrylamide gel electrophoresis
SEM: Standard error of the mean
SLC2A1: Solute carrier family 2 member 1
TCGA: The cancer genome atlas
TME: Tumor microenvironment
UHPLC-QQQ-MS: Ultra-high performance liquid chromatography coupled with triple quadrupole mass spectrometry
β2-AR: Adrenoceptor beta 2
