# Supplementary material for: Chronic stress promotes colorectal cancer progression by enhancing glycolysis through β2-AR/CREB1 signal pathway
Source: Int J Biol Sci. 2023 Apr 2;19(7):2006–19. doi: 10.7150/ijbs.79583 (PMC10158030; doi:10.7150/ijbs.79583)
Supplement: Supplementary file 1 — Supplementary figures and tables. [file ijbsv19p2006s1.pdf]

Supplementary Figure legends

Figure S1. Chronic stress promoted colorectal cancer development. **(A)** The schematic diagram of chronic stress combined with colorectal cancer mouse model. The mice were adaptation for 3 days before subjecting to 14 days chronic restraint stress paradigm, followed with behavioral test, then, MC38 cells were injected subcutaneously. **(B)** The body weights of control and stressed mice under the chronic stress combined CRC procedure were shown (n=6). **(C)** Chronic stress increased the adrenal gland weight. Both sides of adrenal glands from each control and stressed mice were weighed (n=6). **(D and E)** Chronic stress promoted colorectal cells proliferation. Control and epinephrine (Epi, 10 nM) treated SW480 **(D)** and MC38 **(E)** cells were subjected to CCK8 assays. The data are represented as the means ± SEM of at least three independent experiments. *, $p < 0.05$; **, $p < 0.01$; ***, $p < 0.001$.

Figure S2. Chronic stress increased glycolysis in CRC development. **(A)** The medium of tumor isolated cells from stressed xenografts turned yellow faster than that from control xenografts, the indicated cell culture medium color were shown. **(B)** The indicated gene expression levels in control and stress groups cells were determined by RT-qPCR. **(C)** The indicated protein expression levels in control and stress groups cells were determined by western blot. The data are represented as the means ± SEM of at least three independent experiments. *, $p < 0.05$.

Figure S3. Chronic stress activated β2-AR/CREB1 signaling pathway. (A) The mRNA expression levels of 9 adrenoreceptors in CT26, SW480, NCM460 and LoVo cells were detected by RT-qPCR. **(B)** The expression level and localization of CREB1 in control and stressed cells were evaluated by immunofluorescence. **(C)** The phosphorylation of CREB1 in NCM460 and LoVo cells followed by 10 μM epinephrine treatment at indicated time were detected by western blot. **(D)** The effects of ICI 118,551 (10 μM, pretreated 2 h) on epinephrine (10 μM) mediated CREB1 phosphorylation were detected by western blot in NCM460 and LoVo cells. **(E)** The effects of H-89 (10 μM, pretreated 2 h) on epinephrine (10 μM) mediated CREB1 phosphorylation were detected by western blot in NCM460 and LoVo cells.

Figure S4. The correlation of CREB1 and glycolytic enzymes. **(A-C)** The correlation of CREB1 expression with SLC2A1, HK2 and PFKP from TCGA analysis of COAD and CRED were shown.

Supplementary Table legends

Table S1. Primer sequences of glycolytic enzymes and adrenoreceptors for quantitative real-time PCR.

Table S2. Quantitative real-time PCR primer sequences in ChIP assays.

Figure S1

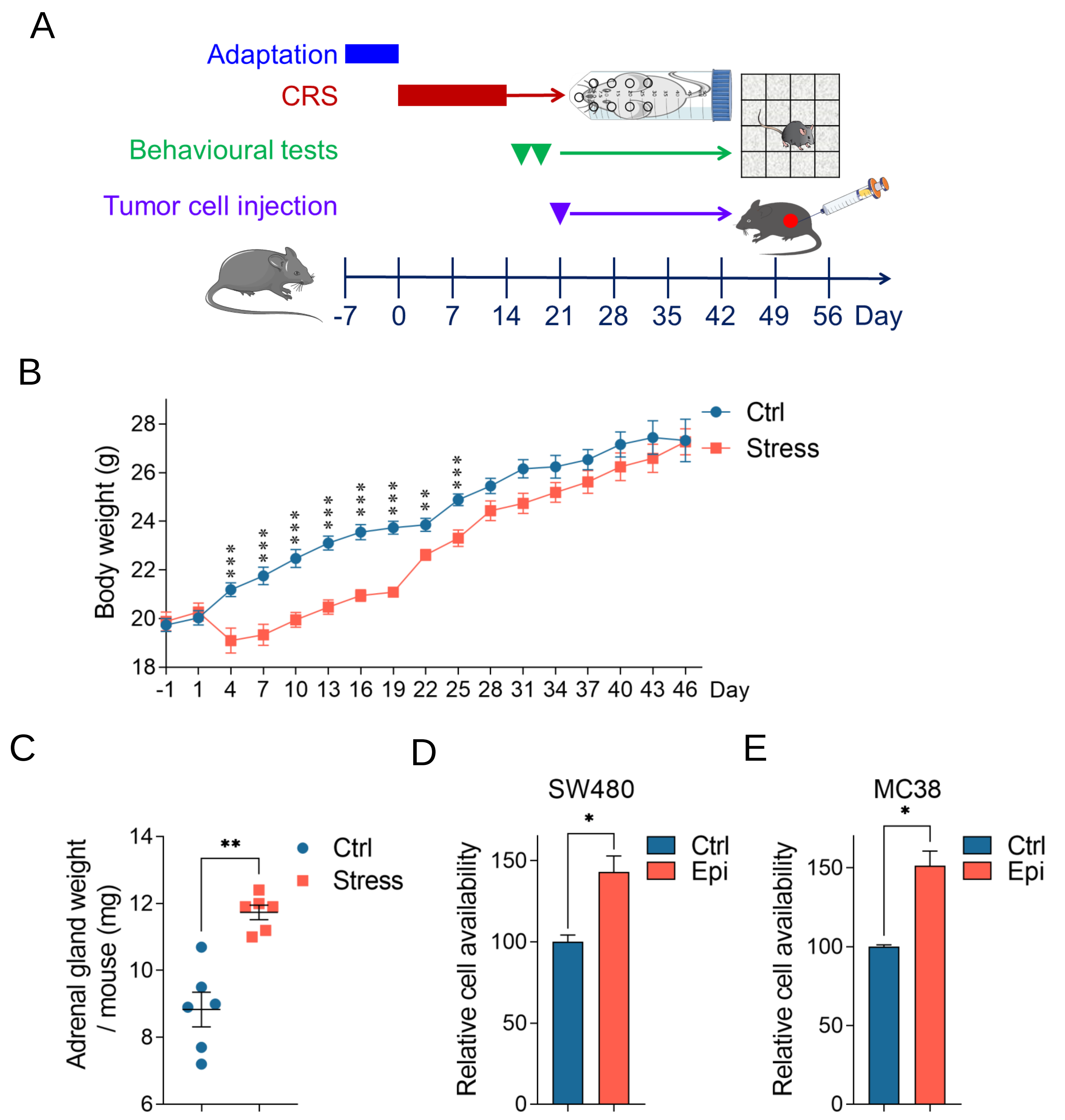

A
Adaptation
CRS
Behavioural tests
Tumor cell injection
-7 0 7 14 21 28 35 42 49 56 Day
B
Body weight (g)
Ctrl
Stress
-1 1 4 7 10 13 16 19 22 25 28 31 34 37 40 43 46 Day
C
Adrenal gland weight / mouse (mg)
Ctrl
Stress
D
SW480
Relative cell availability
Ctrl
Epi
E
MC38
Relative cell availability
Ctrl
Epi

Figure S2

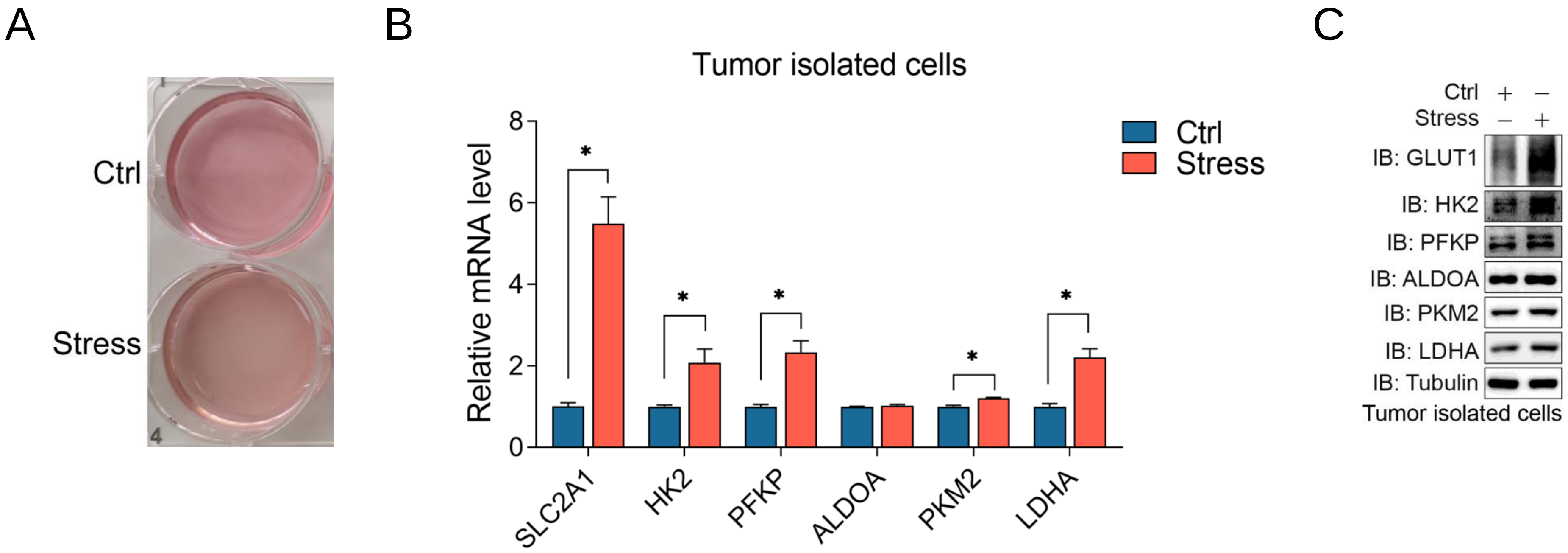
A
Ctrl
Stress
B
Tumor isolated cells
Relative mRNA level
8
6
4
2
0
SLC2A1
HK2
PFKP
ALDOA
PKM2
LDHA
Ctrl
Stress
C
Ctrl + –
Stress – +
IB: GLUT1
IB: HK2
IB: PFKP
IB: ALDOA
IB: PKM2
IB: LDHA
IB: Tubulin
Tumor isolated cells

Figure S3

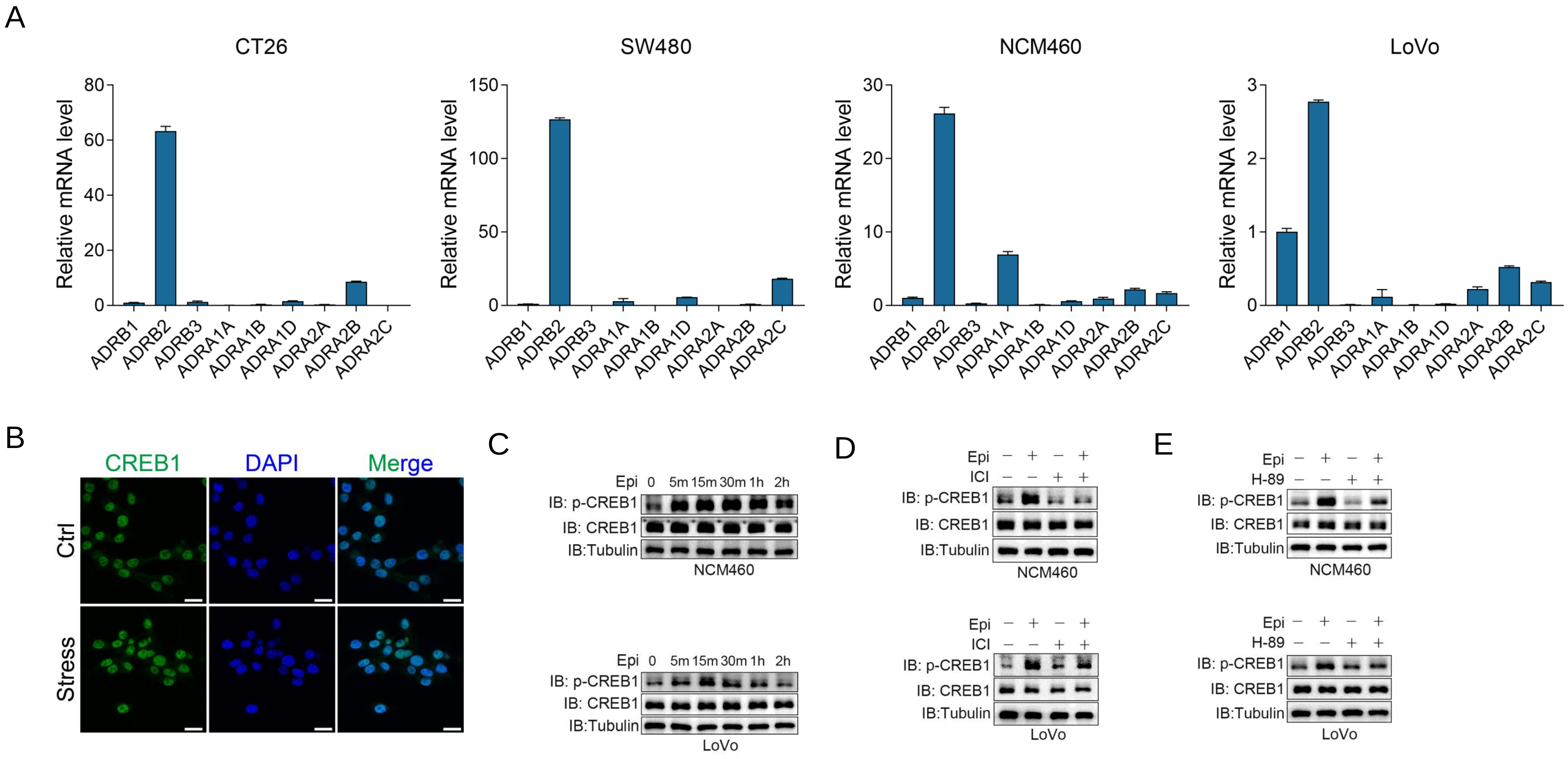
A
CT26
SW480
NCM460
LoVo
Relative mRNA level
ADRB1
ADRB2
ADRB3
ADRA1A
ADRA1B
ADRA1D
ADRA2A
ADRA2B
ADRA2C
B
CREB1
DAPI
Merge
Ctrl
Stress
C
Epi 0 5m 15m 30m 1h 2h
IB: p-CREB1
IB: CREB1
IB:Tubulin
NCM460
LoVo
D
Epi
ICI
E
H-89

Figure S4

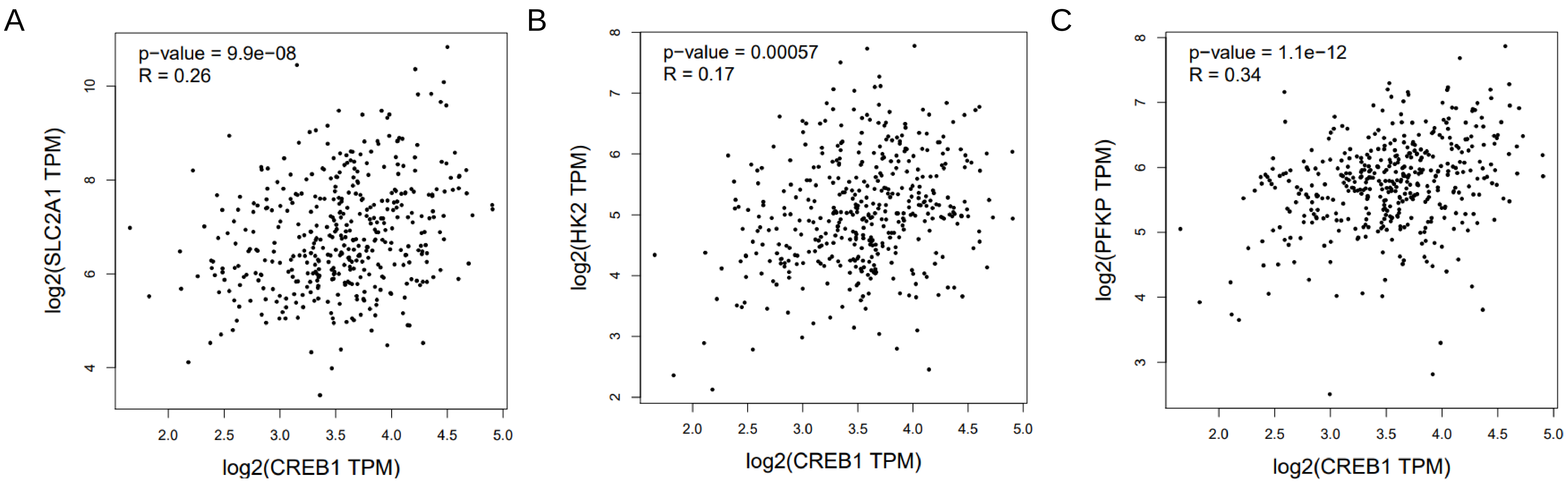
A
p−value = 9.9e−08
R = 0.26
log2(SLC2A1 TPM)
log2(CREB1 TPM)
B
p−value = 0.00057
R = 0.17
log2(HK2 TPM)
log2(CREB1 TPM)
C
p−value = 1.1e−12
R = 0.34
log2(PFKP TPM)
log2(CREB1 TPM)

Table S1		
No.	primer	sequence (5'-3')
1	moACTIN-F	GTACTCTGTGTGGATCGGTGG
2	moACTIN-R	GCAGCTCAGTAACAGTCCG
3	moGLUT1-F	TACACCCCAGAACCAATGGC
4	moGLUT1-R	CCCGTAGCTCAGATCGTCAC
5	moHK2-F	CTGCTTTGGAGATCCGAGGG
6	moHK2-R	GTCTAGCTGCTTAGCGTCCC
7	moPFKP-F	GACGGCTTTGAAGGATTCGC
8	moPFKP-R	CTTTCCAGGTAGGGTGCGTT
9	moALDOA-F	TGGCGCTGTGTGCTAAAGAT
10	moALDOA-R	GCTTCAAGTCATGGTCCCCA
11	moPKM2-F	CATTACCAGCGACCCCACAG
12	moPKM2-R	TCACGGCAATGATAGGAGCC
13	moLDHA-F	GCGTCTCCCTGAAGTCTCTT
14	moLDHA-R	GCTTGATCACCTCGTAGGCA
15	hoACTIN-F	GAGCACAGAGCCTCGCCT
16	hoACTIN-R	GAAGCCGGCCTTGCACA
17	hoGLUT1-F	ACTGTGTGGTCCCTACGTCT
18	hoGLUT1-R	CCGGAAGCGATCTCATCGAA
19	hoHK2-F	GCGTGGACTACTCTTCCGAG
20	hoHK2-R	GCCAGGCAGTCACTCTCAAT
21	hoPFKP-F	ACCAACCTGTGTGTGATCGG
22	hoPFKP-R	TATCGATCTGGCCGTTCCTG
23	hoALDOA-F	AAGATTGGGGAACACACCCC
24	hoALDOA-R	GGATCTCAGGCTCCACGATG
25	hoPKM2-F	CAGAGGCTGCCATCTACCAC
26	hoPKM2-R	GAGGACGATTATGGCCCCAC
27	hoLDHA-F	GCCGATTCCGGATCTCATTGC
28	hoLDHA-R	AGCTGATCCTTTAGAGTTGCCA
29	moADRB1-F	GCCCTTTCGCTACCAGAGTT
30	moADRB1-R	ACTTGGGGTCGTTGTAGCAG
31	moADRB2-F	CAATAGCAACGGCAGAACGG
32	moADRB2-R	TCAACGCTAAGGCTAGGCAC
33	moADRB3-F	TGCTTAGGGAAAAGAGAGCACC
34	moADRB3-R	GCCATAGTGAGGAGACAGGGAT
35	moADRA1A-F	GGCTCTTTCTACGTGCCACT
36	moADRA1A-R	TGACTTGTCGGTCTTGAGGC
37	moADRA1B-F	GCCATCTCCATTGACCGCTA
38	moADRA1B-R	CATTGGGCGCAGGTTCTTTC
39	moADRA1D-F	GCCACTCGCTCAAGTATCCA
40	moADRA1D-R	CAACCTAGTAGCGGTCCCAC
41	moADRA2A-F	AGCTGCAAGATCAACGACCA
42	moADRA2A-R	ACGCTTGGCGATCTGGTAAA
43	moADRA2B-F	TTCCAGCCTCGGCTAAAGTG
44	moADRA2B-R	TTCGGGATCTTCAGGGGTCT
45	moADRA2C-F	GACGCAAGCGGTAGAGTACA
46	moADRA2C-R	GTAGAACGAGACGAGAGGCG
47	hoADRB1-F	TACAACGACCCCAAGTGCTG
48	hoADRB1-R	GTACACGAAGGCCATGATGC
49	hoADRB2-F	CCCTTATCTACTGCCGGAGC
50	hoADRB2-R	CCGTTGCTGGAGTAGCCATT
51	hoADRB3-F	CAGGTGATTTGGGAGACCCC
52	hoADRB3-R	CACTGGTGTTGGCGGTATTG
53	hoADRA1A-F	TTCTGCTCGGGGTGATCTTG
54	hoADRA1A-R	TAGTGCGTGACTGAGTGCAG
55	hoADRA1B-F	CAGAAGCGGCTCATTGAAAGC
56	hoADRA1B-R	CGGCAGCTCCAAGTTTAATGGTC

57	hoADRA1D-F	GACTCAGGTGCCCAGAACTC
58	hoADRA1D-R	CATGCTTGGGGCTGTCTACT
59	hoADRA2A-F	ATCCTGGCCTTGGGAGAGAT
60	hoADRA2A-R	TCTCAAAGCAGGTCCGTGTC
61	hoADRA2B-F	AGATTTGGAAGGGCACCGAG
62	hoADRA2B-R	CACATCCCAGGGCGATACTC
63	hoADRA2C-F	GAACCTCTTCCTGGTGTCGC
64	hoADRA2C-R	CGATCGACGAGGTGCAAAAC

Table S2		
No.	primer	sequence (5'-3')
1	moCHIP-GLUT1-F	CCAGACTGTGGTCAGTAGCC
2	moCHIP-GLUT1-R	TTTTTATAGGACCGCCGCCA
3	moCHIP-HK2-F	CCTCGGATCTCCAAAGCAGA
4	moCHIP-HK2-R	CCAATGAGCACATCCACGTC
5	moCHIP-PFKP-F	TGGACACGCGTTCTGTTAGG
6	moCHIP-PFKP-R	ACCCGGCTATAATCCCTCCA
7	hoCHIP-GLUT1-F	GTTTATAGGACCCCGGCCAT
8	hoCHIP-GLUT1-R	CCTGAGCGAGGCAGTGGTTA
9	hoCHIP-HK2-F	TGCGCACGTCACTGATCC
10	hoCHIP-HK2-R	TAGCTGGGTGACACGATGTT
11	hoCHIP-PFKP-F	CATGGACGCGGACGACTC
12	hoCHIP-PFKP-R	TGGTCAGCACGCCGATG
